# Response Surface Methodology (RSM) Approach for Optimizing the Processing Parameters of 316L SS in Directed Energy Deposition

**DOI:** 10.3390/ma16237253

**Published:** 2023-11-21

**Authors:** Eden Amar, Vladimir Popov, Vyas Mani Sharma, Shir Andreev Batat, Doron Halperin, Noam Eliaz

**Affiliations:** Department of Materials Science and Engineering, Tel Aviv University, Ramat Aviv, Tel Aviv 6997801, Israelvyasmani@tauex.tau.ac.il (V.M.S.); shir.batat@outlook.com (S.A.B.); neliaz@tau.ac.il (N.E.)

**Keywords:** directed energy deposition (DED), laser engineered net shaping (LENS^®^), 316L stainless steel, response surface methodology (RSM), design of experiments (DOE)

## Abstract

Directed energy deposition (DED) is a crucial branch of additive manufacturing (AM), performing repairs, cladding, and processing of multi-material components. 316L austenitic stainless steel is widely used in applications such as the food, aerospace, automotive, marine, energy, biomedical, and nuclear reactor industries. Nevertheless, there is need for process parameter optimization and a comprehensive understanding of the individual and complex synergistic effects of process parameters on the geometry, microstructure, and properties of the deposited material or component. This is essential for ensuring repeatable manufacturing of parts across a single or series of platforms over time, or for minimizing defects such as porosity. In this study, the response surface methodology (RSM) and central composite design (CCD) were employed to investigate the effects of laser power, laser scan speed, and powder mass flow rate on layer thickness, density, microstructure, and microhardness of 316L steel processed by Laser Engineered Net Shaping (LENS^®^) DED. Polynomial empirical prediction models correlating the applied processing parameters and the studied responses were developed.

## 1. Introduction

Additive manufacturing (AM) using powders as feedstock materials has become an attractive alternative for traditional manufacturing processes in a wide variety of industries and applications. The two most important branches of metal AM are Powder Bed Fusion (PBF) and Directed Energy Deposition (DED) [1,2]. Each of these two complimentary manufacturing approaches has its own set of advantages and disadvantages [1,3]. The advantages of DED include, among others, the ability to print either full parts or specific features; perform cladding or repair within a single machine; achieve high deposition rates with compliance for large components; and process multi-materials [4]. Additionally, DED often results in denser and mechanically stronger printed components compared to PBF. The larger chamber size of DED systems allows installation of in situ detection and monitoring instruments, and the operating software is typically more “open” than that of PBF systems This enables investigation of the effect of each processing parameter independently, leading to comprehensive control over the microstructure and repeatability of mechanical properties [5]. Laser Engineered Net Shaping (LENS^®^) is one of the earliest commercialized DED technologies. It was developed by Sandia National Laboratories and licensed to Optomec, Inc. (Albuquerque, NM, USA) in 1997. 

Among various metal DED processes, powder-based feedstock with a laser beam as the energy source is the most extensively used process. In this process, two fundamental factors significantly impact the quality of the components: the energy delivered per second per unit area and the powder feed rate per second per unit area [6]. These factors are significantly influenced by the laser power, laser scan speed (LSS), and powder mass flow rate (PMFR). The global energy density (GED) decides the amount of energy delivered to the material during the deposition process. It plays a crucial role in determining the properties of DED samples. The GED can be calculated as described in Ref. [7]:

(1) $$\mathrm{GED}\left[J/\left(\frac{\mathrm{kg}}{s}{\mathrm{mm}}^{3}\right)\right]=\frac{P}{m\cdot \nu \cdot d_{\mathrm{beam}}^{2}}$$

where *P* is laser power, *m* is PMFR, *v* is LSS, and *d*_beam_ is the diameter of the laser beam. Optimizing the GED in DED helps find a balance between the desired material properties, deposition rate, surface finish, and distortion. It requires careful consideration of various process parameters, such as laser power, LSS, PMFR, working distance, and others, to achieve the desired outcome for specific applications.

316L austenitic stainless steel (SS) is widely used in various applications, including the food processing, aerospace, automotive, marine, energy, biomedical, and nuclear reactor industries [8]. Due to its extensive use and favorable properties, 316L SS is one of the most commonly used alloys in AM techniques, including DED. Svetlizky et al. [5] conducted a comprehensive review of laser-based DED of a wide variety of materials, including 316L SS. The microstructure of AM’ed (including DED’ed) 316L SS is typically anisotropic and depends on the build orientation and heat transfer direction [9,10]. Consequently, the mechanical properties are typically anisotropic too [5]. Guo et al. [11] reported that the tensile properties of DED’ed 316L SS samples with the layer scan direction perpendicular to the tensile test axis were better than those of samples whose layer scan direction was parallel to the tensile test axis. Zheng et al. [12] found that microstructure evolution during LENS^®^ processing of 316L SS is complex due to the high thermal gradient and dynamic flow in a fast-moving melt pool with associated rapid solidification and the presence of layer-by-layer deposition. The laser focus position plays a critical role in determining the surface quality of DED-deposited components. Selecting a laser under focused conditions can effectively prevent the accumulation of un-melted powder on the sidewalls of deposited sections.

Despite the abundance of research focused on DED of 316L SS, particularly in exploring various microstructural facets and material properties (Table 1), there is a noticeable scarceness of studies that specifically address process parameter optimization methodologies and systematic elucidation of the interdependencies between process parameters and pivotal material properties. This is essential also for repeatable manufacturing of parts across a single or series of platforms over time or for minimizing defects such as porosity and achieving the desirable mechanical properties [13].

For instance, Aversa et al. [14] found that higher laser power yields denser samples with less pores. The authors concluded that lower laser power resulted in fine microstructures due to higher cooling rates. However, despite the increased porosity in sample processes at lower laser powers, these samples exhibited superior tensile properties and inferior dislocation free paths compared to the denser ones processed under high laser power. Kumaran et al. [15] showed a correlation between laser power and the porosity and microhardness of DED-processed 316L SS. At low laser power (400 W), higher LSS resulted in more porosity and lower hardness values due to the presence of un-melted powder particles. At medium laser power (600 W), the hardness increased due to finer grains and no un-melted powder particles were detected. It was also reported that DED-processed 316L SS exhibited higher microhardness than PBF-processed steel. In tensile tests, DED-processed 316L SS displayed higher elongation, the same ultimate tensile strength (UTS), lower yield strength (YS), and higher strain hardening compared to the PBF-processed steel. Era et al. [16] investigated the effects of process parameters (laser power, LSS, energy density, and layer thickness) on the tensile behavior of DED’ed 316 SS components. Layer thickness had the most significant impact on UTS, increasing layer thickness and leading to an increase in UTS. Laser power had the lowest impact on UTS; increasing laser power did not affect the UTS significantly. On the other hand, an increased LSS or a decreased laser energy density resulted in a decrease in UTS. Yang et al. [17] found that for DED’ed 316L SS components, low LSS resulted in less porosity due to melting of the previous layer and complete powder melting. LSS affected the grain size too—slower LSS yielded larger grains due to lower cooling rate and vice versa.

**Table 1:** Summary of process parameters used in AM of 316 SS.

AM Technology	Alloy	Laser Power, W	Laser Scan Speed, mm/min	Hatching, mm	PMFR, g/min	Layer Thickness, mm	Analysis	Ref.
DED-LENS	316	100/200/250	510/762/1020	0.254	7/10/13.5	0.381	Relation between dimensions, porosity, and mechanical properties.	[18]
PBF	316L	400/600	300/500/				The mechanical and microstructural properties of sandwich structure fabricated by combining PBF and DED.	[15]
DED-LENS		800	700		2/3/4			
DED	316L	2000	500	3		1	Evaluation of microstructure, mechanical properties, and machinability of AISI 316L stainless steel.	[11]
DED	316L	400/700	120/600	-	10/30		Microstructural analysis, mechanical properties testing, including hardness and friction.	[19]
DED-LENS	316L	250/328/270–516	1014	0.25	28	0.38	Evolution of dimensional and surface quality, microstructure, defects, and mechanical properties.	[12]
DED-LENS	316L	1000	360/480/600/720	1		0.5	Scanning strategies, microstructure characterization, density, hardness, tensile properties.	[17]
DED-LENS	316	600/700/800/900/1000	480/540/600/660/720		5	0.54	The effects of laser power, scan speed, energy density, and layer thickness on the material’s tensile strength.	[16]
DED	316L	1400/1800/2000	360	-	15.1		Optimization of the surface evenness and deposition efficiency, design of the mathematic model to predict the quality (dilution) of DED-built parts as a function of processing parameters such as laser power, PMFR, and LSS.	[7]
DED-LENS	316L	645	127–1143	-	3.34–7.5	-	Microstructure, microhardness, and porosity.	[20]
DED-LENS	316L	360	1008	0.39	10	0.25	Physical metallurgy, tensile properties, and Vickers microhardness.	[21]
DED-LENS	316L	360	510		5.4	0.5	Mechanical properties and microstructure correlation with the inter-layer time intervals .	[22,23]
DED	316L	900	900				Effect of scanning strategies on microstructure and mechanical properties.	[24]
DED	316	100	400/500/600		8		Effect of parameters on microstructure, microhardness, wear resistance.	[25]
DED-LENS	316L	400	900	0.45		0.3	Microstructure, microhardness, corrosion resistance.	[26]
DED	316L	1000	360		4.68		Microstructure, mechanical properties (hardness and tensile) of thin-walled parts.	[27,28]
DED-LENS	316L	380			6.3		Microstructural analysis and damage evolution in compression tests.	[29,30]
DED	316L	34.3/45.2	24/30/36		2.46/2.81/3.09		Microstructural analysis, tensile properties.	[31]
DED	316L	200/375	138/240/1524		10		Effect of process parameters on microstructure.	[32]
DED	316L	417	850			0.25	Fracture analysis of tensile tested parts.	[33]
DED	316L/TiB_2_	1000/1200/1400	200/400/600	-	5.44/7.85/9.95	1	Effect of laser power, scan speed, and hopper speed on the microhardness and density of the composite material.	[34]
DED	316L/WC	570	290	-	23.4		Microstructural analysis and thermal model design for prediction of the thermal history and the melt pool depth.	[35]
DED	316L/Inconel 718	250–900	50	-	4	1.2	Evaluation of composition gradients, functionally gradient material (FGM) geometry, and the FGMs build process. Effects of processing parameters, such as laser power, PMFR, LSS, and z-axis movement, on the quality of the FGM.	[36]

The *primary motivation* of this research was to address the aforementioned gap by demonstrating the effective utilization of the Design of Experiments (DOE) methodology for optimizing process parameters specifically for 316L SS and Optomec’s LENS^®^ machine. It should be noted that the term DOE has occasionally been used in a confusing manner. For example, in Ref. [20], LENS^®^ was initially employed to process 90 single beads at a constant laser power of 645 W, which was selected because it was approximately the center of the 300–1000 W range used in several previous studies. While a matrix of PMFR-to-LSS ratios (i.e., linear mass densities) was drawn, it was not based on statistical tools. The reason for selecting a specific limited number of single beads for characterization was not justified, and no mathematical formulation of interdependencies between process parameters was drawn. Multilayer coupons were deposited in the second step using the same process parameters as for selected single beads solely to demonstrate the consistent microstructure with a low level of porosity and microhardness equal to or better than that of wrought 316L SS [37]. The present study is substantially different, as will be explained below.

In this study, the Central Composite Design (CCD) approach was employed. CCD is a widely used DOE approach. It falls under the category of response surface methodology (RSM), wherein a second-order polynomial equation is fitted to the experimental data derived from the design matrix [6]. RSM is a statistical method for developing mathematical models that describe the relationship between process parameters and the responses of interest [38]. For example, in Ref. [39], RSM was used to establish the relationships between the energy input (laser power, LSS, layer thickness, and hatch distance) and the quality features of thin-walled parts fabricated using selective laser melting (SLM). By employing RSM, the researchers were able to systematically reveal the role of each process parameter on the thin wall’s primary properties, such as track width, surface roughness, and hardness, and additionally were able to find the optimal manufacturing conditions for high-quality thin-walled parts.

Among the advantages of RSM are its ability to identify the significant factors affecting the response, to model the response surface and optimize the process parameters, and to reduce the number of experiments required to achieve the desired quality [40,41]. The design matrix in CCD comprises three types of experimental runs: factorial points, axial points, and center points. Factorial points encompass a full factorial design, considering all possible combinations of high and low levels for each factor. Axial points are strategically located at a distance α from the center point to estimate the response surface curvature. Center points serve to gauge experimental error and the pure error attributed to system variability. The outcome of a CCD analysis is a mathematical model that describes the relationship between the process variables and the responses. Subsequently, this model can be utilized to forecast the optimal process parameters required to attain a desired response, e.g., specific mechanical properties or a target density level.

The CCD method is one of several DOE methods applied in the optimization of AM processes. The Taguchi method is another notable DOE approach used for process parameter optimization, albeit with some key distinctions [42]. The Taguchi method is primarily geared towards achieving optimal process parameters that minimize variation in the response variable. It leverages orthogonal arrays to explore the process parameters efficiently, often requiring a relatively small number of experiments. This method is particularly suitable for processes that exhibit stability and are not susceptible to significant fluctuations. On the other hand, CCD is better suited for processes that may be less stable, potentially exhibiting greater variability over time. While both CCD and the Taguchi method offer valuable utility for process optimization, CCD exhibits a particular aptitude in the context of AM. Its distinctive strength lies in its capability to proficiently model the intricate and multifaceted relationship between process parameters and responses. This makes CCD exceptionally well-suited for the intricate and often less stable characteristics of AM processes, where a comprehensive understanding of these intricate relationships is imperative for the attainment of repeatable results. CCD enables an extensive exploration of process variables with a relatively small number of experimental runs. By incorporating axial and center data points alongside full or fractional factorial data points in the experimental matrix, CCD enhances the efficiency of the investigation.

The *ultimate goal* of this study was to examine the effect of selected parameters (laser power, LSS, and PMFR) on selected responses (layer thickness, steel density, microstructure, and microhardness) and to establish a reliable set of process parameters that would enhance the repeatability and overall performance of the DED process, thus contributing to the advancement of laser- and powder-based AM technologies. 

## 2. Materials and Methods

### 2.1. Powder Characteristics

**Figure 1:**
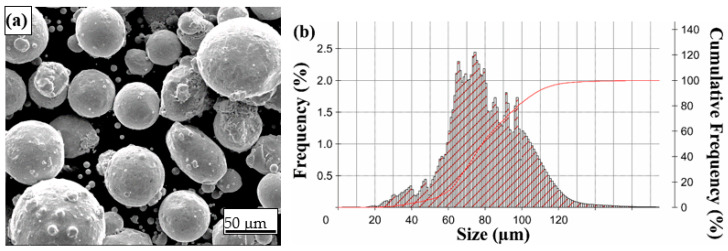
(**a**) SEM image revealing the 316L powder particles’ surface morphology. (**b**) PSD of the 316L powder: *d*_10_ = 56.68 µm, *d*_50_ = 78.45 µm, *d*_90_ = 107.28 µm.

Gas atomized 316L SS powder (Sino-Euro Materials Technologies, Xi’an Co., Ltd., Xi’an, China) was used as a powder feedstock. The powder particle size range was 56–107 μm, within the range of 44–150 μm recommended by Optomec for LENS^®^ processing. The chemical composition, powder morphology, and particle size distribution (PSD) are shown in Table 2 and Figure 1.

### 2.2. LENS^®^ DED

The samples were fabricated using Optomec’s LENS^®^ 3D Hybrid 20 Controlled Atmosphere System at Tel-Aviv University’s Additive Manufacturing R&D Center. The system is equipped with 2 kW Nd:YAG laser (IPG Photonics, Inc., Oxford, MA, USA). 316L SS plate with dimensions of 100 × 100 × 10 mm was used as the base plate. 

In the DED process, various parameters directly affect the microstructure, physical, and mechanical properties of the deposited material [43,44]. In this study, laser power, LSS, and PMFR were selected as the dominant processing parameters based on prior experience and relevant literature [6,20,43]. Both single-layer and cubic (10 × 10 × 10 mm^3^) coupons were manufactured according to the CCD matrix (see Table 3). In all cases, the laser spot diameter was 0.5 mm.

The powder flow was assessed by weighing the powder for a given time period under a known argon carrier gas flow rate (liters per minute, LPM) and powder feed rate (revolutions per minute, RPM). PMFR is then the amount of powder per minute that is supplied to the melt pool area. The PMFR was measured using a container holding the powder for an exact time and a scale. According to the DOE, PMFRs of 10, 20, and 30 g/min were employed. Experimentally it was determined that these PMFR values could be achieved with a carrier gas flow rate of 4 LPM and powder feed rates of 7, 15, and 25 rpm, respectively.

### 2.3. Design of Experiments—CCD

**Figure 2:**
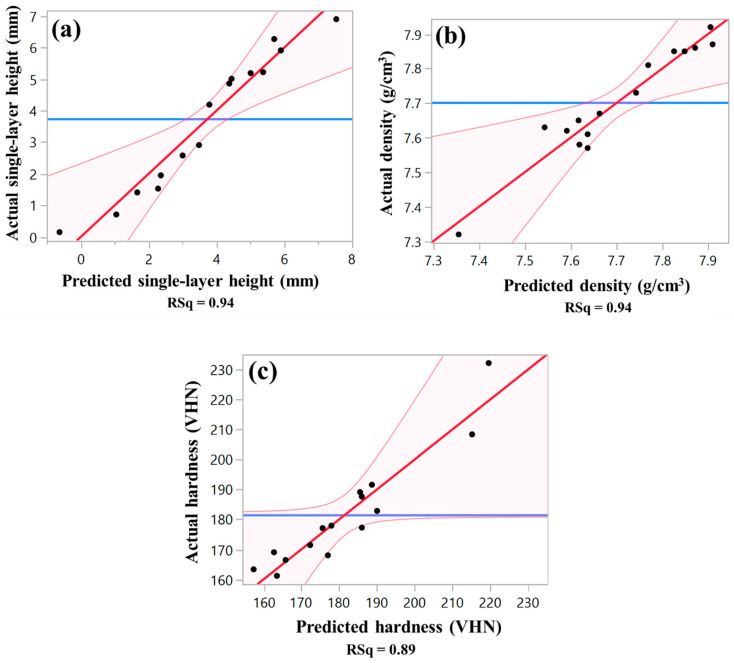
Comparison between the predicted and actual data. (**a**) Single-layer thickness, (**b**) density, (**c**) microhardness responses. Blue line shows the average value of the response, while red line shows the fitted line. The black dots represent the values of predicted and obtained responses on the *x* and *y* axes. The pink area shows the intersection of the average *y*-axis response and fitted line.

A three-factor CCD was generated and analyzed using JMP 17 Pro data analysis software (SAS Institute Inc., Charlotte, NC, USA). The CCD experimental matrix comprised 16 parameter combinations, with 8 factorial points, 6 axial points, and 2 replicated center point experiments. These points are denoted by a three-character pattern of numbers, letters, and symbols (Patterns in Table 3, dots in Figure 2). The points were added to assess the model’s error, validity, and reproducibility [44]. 

Table 3 presents the experimental matrix along with the corresponding analyzed response data. The implemented RSM enables the determination of the influence of independent input processing parameters on the output geometrical characteristics and enables the establishment of a prediction model for the studied responses. The response prediction model is fitted to the input variables using the following second-order polynomial equation [45]:

(2) $$Y=\alpha_{0}+\sum_{i=1}^{n}\alpha_{i}r_{i}+\sum_{i=1}^{n-1}\sum_{j=i+1}^{n}\alpha_{ij}r_{i}r_{j}+\sum_{i=1}^{n}\alpha_{ii}r_{ii}^{2}+\varepsilon$$

Equation (2) represents the predicted response variable *Y,* where *r_i_* and *r_j_* denote the independent factors, α*_0_*, α*_i_*, α*_ij_*, and α*_ii_* are the model coefficients, and *ε* indicates the prediction model error, which accounts for independent experimental error. By utilizing RSM, a process parameters map and empirical formula were established to predict the direct effect and relationship between the studied DED process parameters (Table 4) and between the analyzed responses of the as-deposited 316L SS (Table 2) within the range of factor levels examined (Table 4). Statistical analysis was conducted with a significance level of less than 5% (*p* < 0.05, *t*-test) and a lack of fit level *p* > 0.05 (*F*-test). The experimental data underwent two stages of fitting and analysis. In the first stage, all effects in Equation (2) and normalized versions of the factors were considered. Non-significant effects were then eliminated based on defined significance criteria for their impact on all studied responses. In cases of effect heredity, first-order effects were retained if the factor was involved in a second-order effect, even if they did not meet the model’s significance criteria. In the second stage, the resulting reduced model was fitted without the normalization of factors. Table 4 provides the experimental matrix with the variable factors and corresponding factor levels used for the deposition of 316L SS specimens. The model responses were selected as single-layer thickness (*h*), alloy density (*ρ*), and microhardness (H). 

### 2.4. Samples Preparation

The specimens’ single-layer heights were measured using a digital micrometer. For each set of parameters, single-layer height measurements were taken of three samples. The results are presented in Table 3. Samples for density and microhardness measurements were removed from the build plate using electrical discharge machining (EDM)(Mitsubishi MV1200R Connect, Tokyo, Japan). After cutting, the density of the samples was measured using the Archimedes principle, following ASTM B962-17 standard [46]. An analytical BA 210 S balance (Sartorius AG, Göttingen, Germany) with 0.1 mg readability and density determination kit was used. The samples were weighed in deionized (DI) water at room temperature. Subsequently, the cut samples were mounted in epoxy in orientations parallel and perpendicular to the build direction. Mounted specimens were mechanically ground on 320, 600, 800, 1200, 2000, and 4000 SiC abrasive papers, followed by mechanical polishing using diamond suspensions (9, 3, and 1 µm). For final polishing, colloidal silica suspension (0.2 µm) was used. Vickers microhardness tests were performed on both the parallel and perpendicular planes at a load of 200 g and dwell time of 15 s. For microstructural characterization, polished samples were chemically etched using Kalling’s reagent II (5 g CuCl_2_ + 100 mL HCl + 100 mL ethanol). Microstructure characterization was performed using an optical microscope. Further analysis was conducted using scanning electron microscope (SEM, Quanta 200 FEG ESEM, ThermoFisher, Waltham, MA, USA) under high-vacuum conditions. X-ray diffraction (XRD) at room temperature was performed for phase identification. The XRD patterns were acquired by AD8 ADVANCE diffractometer with a Bragg–Brentano geometry (Bruker AXS, Madison, WI, USA) and Cu-Kα radiation source. A linear position sensitive device (PSD) detector (LYNXEYE XE-T) was used, with an opening of 2.94°. Data points were acquired at increments of 0.02° and acquisition time of 0.25 s. The scan was within the range of 2θ = 40–100°.

## 3. Results and Discussion

Successful fabrication of 316L single-layer and bulk samples using LENS^®^ DED on 316L SS substrate was achieved, following the design specified in the three-factor rotatable CCD experimental matrix. It is important to note that as with most model-building processes, a “bias/variance” tradeoff exists. Inclusion of more terms reduces bias but increases variance as more extensive term estimation strains the data, leading to higher variance. Such tradeoffs are especially noticeable in ill-posed problems where not all terms can be fitted, necessitating effect selection or regularization. Even in non-ill-posed cases, effect selection induces regularization, reducing variance but potentially introducing bias.

In this study, second-order polynomial regression prediction models were developed using the least-square method to predict the as-deposited 316L single-layer height (*h*), as well as the density (*ρ*) and microhardness (*H*) of bulk samples. These prediction models, expressed as presented in Equations (3)–(5), encompass both quadratic and linear terms:

(3) $$h=0.95+67.02\times 10^{-4}P-60.35\times 10^{-4}\nu +0.14m$$

(4) $$\begin{array}{ll} \rho = & 8.52-2\times 10^{-4}P+9.62\times 10^{-4}\nu -11.25\times 10^{-3}m+2.03\times 10^{-6}P\;\nu +\\ & 5.05\times 10^{-5}P\;m+12.4\times 10^{-4}m^{2} \end{array}$$

(5) H=193.46+0.18 ν+0.33 m−0.006 ν m

Note that Equations (3)–(5) are only valid within the range of variable factor levels investigated in this study, using input factors with the corresponding units specified in Table 4.

**Figure 3:**
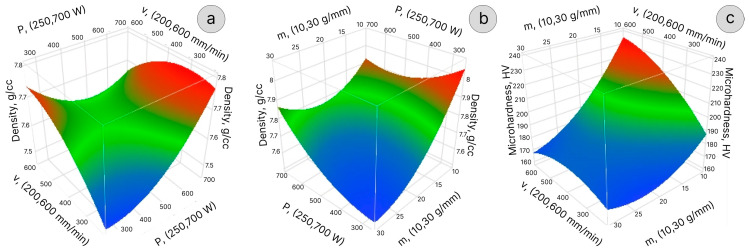
Response surface plots: (**a**) the predicted density as a function of laser power and LSS, (**b**) the predicted density as a function of PMFR and laser power; (**c**) the predicted microhardness as a function of PMFR and LSS.

**Figure 4:**
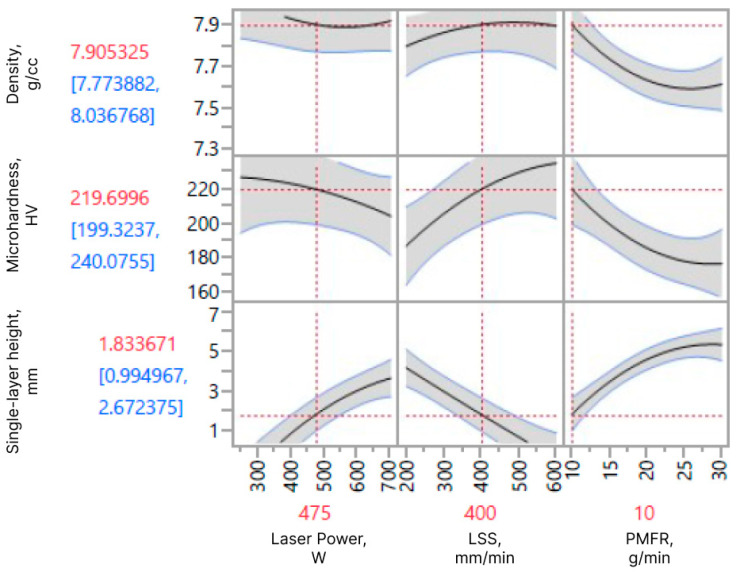
A summary of the effects of process parameters on the single-layer height response, material density response, and microhardness response. The data in blue represent the 95% confidence of the response. Laser power vs. response graph is plotted at *v* = 400 mm/min and *m* = 10 g/min. LSS vs. response graph is plotted at *p* = 475 W and *m* = 10 g/min. PMFR vs. response graph is plotted at *p* = 475 W and *v* = 400 mm/min.

Figure 3a–c depicts the 3D response surfaces demonstrating the influence of the variable factors on the sample density and microhardness prediction at various factor combinations. The sample’s density mapping shows that the density was maximized by setting a low PMFR while keeping the other variable factors at the center point (see Table 3). Figure 3c shows the maximized microhardness achieved when keeping the PMFR at the lowest level and LSS at the highest level. Moreover, as can be seen from Figure 4, the effect of laser power on the microhardness is less significant compared to the other parameters included in the prediction model (e.g., PMFR).

As evident from Equation (1) and Figure 4, PMFR has the most significant effect on the single-layer height, aligning with the experimental findings. Table 5 provides a comprehensive summary of the fit and lack-of-fit analyses for the empirical prediction models, with a significance level set at 95%. This analysis evaluates the degree of correlation between the predicted and actual measured data, determined by the regression coefficient (*R*^2^). The *R*^2^ values for the developed prediction models are as follows: 0.94 for the single-layer height response, 0.94 for the material density response, and 0.89 for the microhardness response (see Figure 2). These values indicate the fit quality and demonstrate the effectiveness of the models in capturing the relationship between the investigated variables.

Previous studies have indicated that a model fit is deemed satisfactory when the *R*^2^ values surpass 0.80 [43,47]. In this study, the developed models for all responses, namely single-layer height, density, and microhardness, convincingly meet this criterion. Moreover, the adjusted *R*^2^ values (adj-*R*^2^ in Table 5) further corroborate the validity of the developed models for the studied responses, with a discrepancy of less than 0.2 observed between the adj-*R*^2^ and the obtained *R*^2^ for each response prediction model [48]. This confirms the reliably of the model. The lack-of-fit analysis serves to assess the validity and accuracy of the developed prediction model, taking into account either the center-point replications (pure error) or the entire model (total error). In the case of all models, the lack-of-fit test reveals no evidence necessitating more complex prediction equations (*p* > 0.05). It is important to note that the response surface prediction model remains valid within the range of factor levels specified in Table 4. However, beyond these levels, the model’s predictions might not be valid, thereby necessitating the inclusion of additional data points [43,47].

Based on the designed prediction models, response surface curves were constructed for each response to depict the relationships between the independent variable factors and the investigated responses at their respective levels. Figure 4 provides an overview of the effects on the single-layer height, steel density, and microhardness responses, specifically at the middle levels of each factor.

### 3.1. Response Surface Analysis of the Single-Layer Height

The first set of printed samples was prepared according to DOE parameters (Table 3) for single-layer printing. In DED, the single-layer print is a crucial step in developing process parameters since it helps to obtain the optimal layer thickness settings. Layer thickness in DED differs from its meaning in PBF. In PBF, layer thickness is the distance the working table goes down layer by layer. Thus, if this distance is 50 µm, the table goes down to 50 µm and the rake deposits a new powder layer according to this distance. In DED, layer thickness is the distance that the depositing head rises. Under said conditions, the thickness of the built layer can be either lower or higher than the set layer thickness parameter. This variation depends on the distance between the deposition head and the substrate and then the sample. If this distance is too high and continues to increase, it causes extremely low energy input, resulting in low sample expansion. Conversely, when the distance between the deposition head and substrate/sample is smaller, the deposited powder layer might have a greater thickness. This will reduce the clearance between the head and the printed part, potentially causing a failed build or even machine damage.

**Figure 5:**
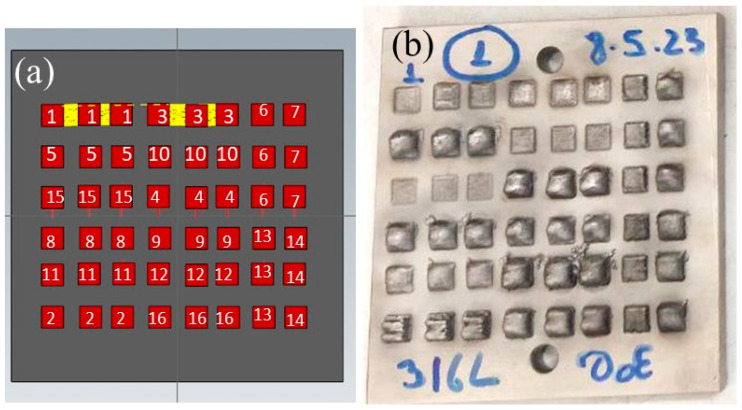
DED samples produced for single-layer height measurements. (**a**) CAD-file image with the marked numbers of the process parameter combinations. (**b**) Printed single-layer samples.

Visual inspection of the single-layer trials reveled that certain samples, for example, those produced by parameters #10 and #15, exhibited insufficient supplied energy to achieve the desired layer thickness (see Figure 5). On the other hand, samples #5, #12, and #16 exhibited excessive height, with single layers measuring 6.265, 6.900, and 5.186 mm thick, respectively. These observations highlight the importance of carefully optimizing the process parameters to ensure successful deposition and meet the required specifications.

Another important parameter is GED. As shown in Table 4, different combinations of laser power, LSS, and PMFR can yield nearly identical energy densities (see samples #1, 3, 4, 10, and 12). Energy density directly influences the material properties of the deposited samples. Higher energy density can result in increased melting and fusion of the material, leading to denser and more homogeneous microstructures, while lower energy density may result in incomplete melting or poor bonding between the layers, leading to porosity or compromised mechanical properties. The energy density is determined by the cooling rates and thermal gradients within the deposited material. Rapid cooling rates associated with higher energy densities can result in higher residual stresses and increased sample distortion. Controlling energy density during DED can help manage the residual stress levels and minimize distortion [49]. Energy density also affects the build rate and productivity of the DED process. Higher energy densities typically result in faster melting and higher deposition rates, reducing lead time. However, it is essential to balance the energy density with other factors such as heat dissipation, material properties, and process stability to ensure quality and reliability [7]. The examples in this study demonstrate that finding the optimal energy density does not necessarily mean finding the optimal processing parameters. 

Single-layer height measurements were carried out on all specimens, see Table 3. Sample #15 exhibited negligible growth with a height of only 0.150 mm. This limited growth can be explained not only by the low energy density (0.6 × 10^6^ [J/(kg·mm^3^/s)]) but also by the relatively low PMFR. In contrast, sample #13 had the same laser power and LSS but higher PMFR, and thus had good expansion of the layer height. Samples #5, 12, and 16 had the highest layer height (6.265, 6.900, and 5.186 mm, respectively). Notably, this order does not correspond linearly with the order of energy density values (5.04 × 10^6^, 1.68 × 10^6^, and 0.57 × 10^6^ [J/(kg·mm^3^/s)], respectively). Samples #5 and #12 were deposited using high laser power, low LSS, and different PMFR (10 and 30 g/min, respectively). This explains the lower energy density observed for sample #12 (*m* value is found in the denominator of the energy density calculation). Samples #4, 7, 8, 9, and 14 also exhibited excessive single-layer heights.

It has been noted that samples with high PMFR and low GED, such as samples #2, 13, 14, and 15, failed due to insufficient energy for the substitutional amount of material deposited. This results in weak adhesion, lack of homogeneity, and delamination between layers. These findings correspond with previous data indicating that PMFR values higher than 10 g/min are not recommended for steels [50]. Deposition of sample #15 was tried twice—once as a single layer and once as bulk. However, due to the combination of low PMFR and laser power with high LSS, growth was unsatisfactory. Although it was possible to measure the effect of parameters on the single-layer height, the bulk sample failed. Lower LSS could improve the specimen’s growth, as demonstrated by sample #10. Another factor contributing to the limited growth of sample #15 could be the distance between the nozzle and the substrate, also known as the “stand-off distance” or “working distance” [50]. The stand-off distance can influence the size and shape of the melt pool. A smaller stand-off distance typically leads to a smaller and more focused melt pool, while a larger stand-off distance may result in a wider and more spread-out melt pool. The choice of stand-off distance depends on the desired melt pool characteristics for the specific application, e.g., for obtaining the desired part resolution or controlling the heat-affected zone (HAZ). In the case of sample #15, the first layer was successful due to relatively adequate working distance; however, this distance was not suitable for bulk sample manufacturing and resulted in failure due to the continuously increasing stand-off distance.

Figure 2a shows the correlation between the actual and the predicted single-layer height. From Figure 4 it can be seen that increasing laser power and PMFR leads to an increase in the single-layer height, whereas increasing LSS at constant laser power and PMFR results in a reduction in the single-layer height. DOE results for the single-layer height response are tabulated in Table 6. It provides the parameters’ effect estimation and their significance level, considering all linear and quadratic factor interactions that influence the single-layer height response. The type of parameter effect, whether negative or positive, on the single-layer height response is also provided. It is evident that only the first-order parameters of the laser power, LSS, and PMFR are statistically significant (*p* < 0.05); other combinations have insignificant impacts and were thus excluded from the analyzed single-layer height prediction model. These findings are in accordance with previous reports [43,50] that the deposit height is primarily influenced by the feed rate and LSS. 

### 3.2. Response Surface Analysis of the Density

**Figure 6:**
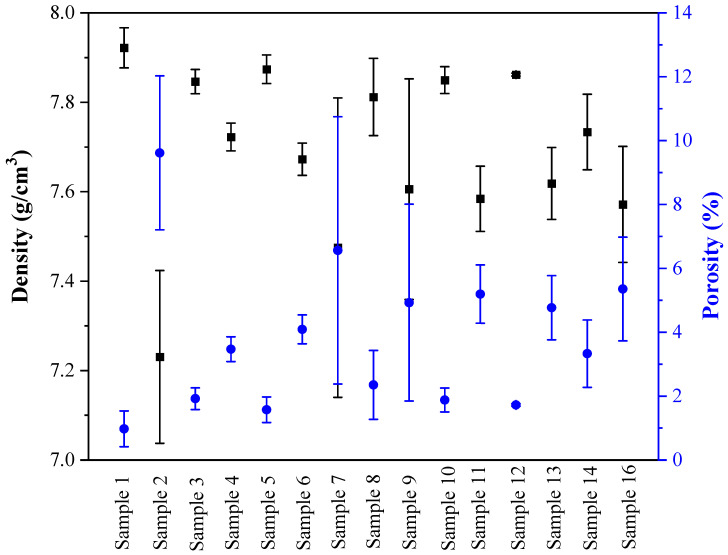
Density and porosity variations in 15 out of 16 parameter sets (sample #15 failed).

In Table 3 and Figure 6 it can be seen that parameter set #1 achieved near-theoretical (~99%) density. In this study, the bulk density of 316L SS was taken as 8.00 g/cm^3^ [51]. Combinations #3, 5, 8, 10, and 12 yielded densities exceeding 98%, while all other combinations yielded densities between 94–97% of the theoretical density. Figure 6 also reveals that denser samples were associated with a smaller variance of density values (see #1, 3, 5, 10, 12). As a result, it is anticipated that the corresponding mechanical properties would be superior as well. Set #2 yielded the lowest density value. This may be attributed to the low energy input. All samples with GED below 0.9 × 10^6^ [J/(kg·mm^3^/s)] exhibited bulk densities less than 98%. As GED increased to the approximate level of 1.7 × 10^6^ [J/(kg·mm^3^/s)], the corresponding densities increased. Figure 2b shows the correlation between the predicted and actual density. Figure 4 presents that an increase in PMFR resulted in lower density. Laser power and LSS seem to have a synergistic effect on the density; varying only one of them does not result in a clear trend in density values (see Table 7). Table 7 summarizes the parameters’ effect estimation and their significance level, considering all linear and quadratic factor interactions that influence the density response. The type of parameter effect (negative or positive) on the density response is also presented. It is evident that the first- and second-order of laser power are insignificant (*p* > 0.05); hence, they were excluded from the analyzed density prediction model. The same applies to the first- and second-order parameters of the LSS and to the combination of LSS with PMFR. Among the significant parameters, the PMFR shows the most significant (negative) effect on the density response. This is expected as an increased PMFR leads to overgrowth and the formation of pores and lack-of-fusion.

### 3.3. Response Surface Analysis of Microhardness

**Figure 7:**
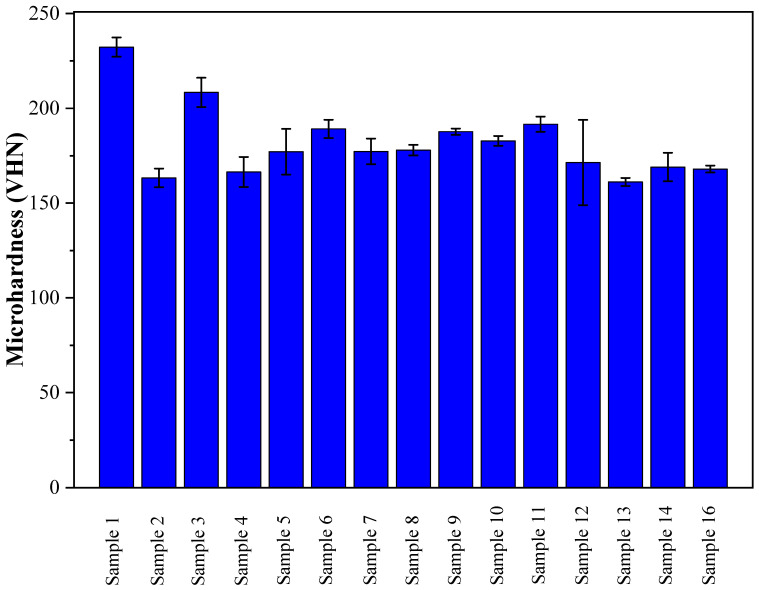
Microhardness values of 316L on a plane perpendicular to the build direction.

Figure 7 shows the results of microhardness measurements on a plane perpendicular to the build direction for all DOE parameter sets. It can be seen that the microhardness of samples #1 and #3 is the highest, reaching 232 and 208 VHN, respectively. The maximum microhardness value (232 ± 6 VHN) was obtained for parameter set #1, which represents the optimal combination of the laser power (475 W), LSS (400 mm/min), and PMFR (10 g/min).

The microhardness of the samples produced with an optimal set of parameters is of the same order as for wrought 316L SS, or even slightly higher [37].

Figure 2c shows the correlation between the predicted and actual microhardness. It can be seen from Figure 4 that an increase in PMFR results in a reduction in microhardness. The laser power had less effect on microhardness, whereas the increase in LSS resulted in an increase in microhardness. Table 8 shows the parameters’ effect estimation and their significance level, considering all linear and quadratic factor interactions that influence the microhardness response. The type of parameter effect (negative or positive) on the microhardness response is also shown. As evident, the second-order parameters of the PMFR, LSS, and laser power are insignificant (*p* > 0.05); hence, they were excluded from the analyzed microhardness prediction model. The same applies to the first-order parameter of the laser power and to the combinations of the laser power with either PMFR or LSS. Among the significant parameters, the PMFR shows the most significant (negative) effect on the microhardness response. As explained above, the reduced microhardness can be attributed to the porosity and lack-of-fusion in samples deposited under high PMFR.

### 3.4. Microstructural Characterization

**Figure 8:**
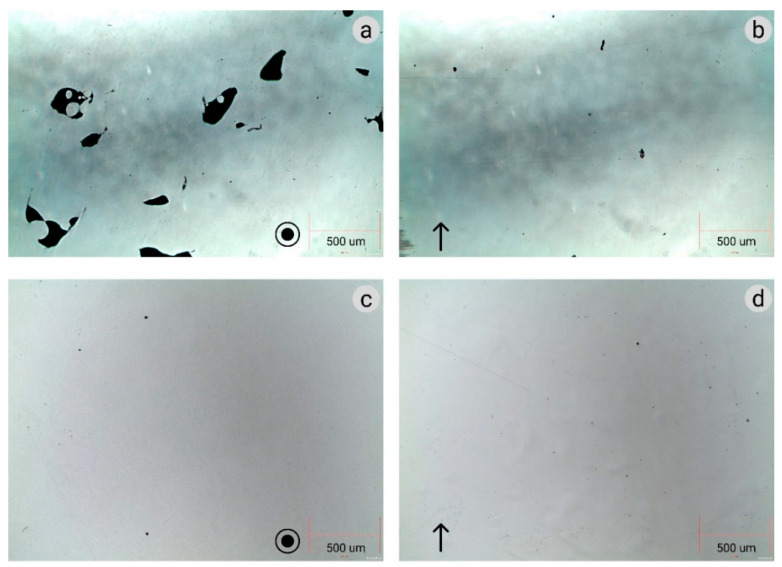
Light microscope images of unetched samples. (**a**,**b**) Set #2, (**c**,**d**) set #1.

Figure 8 shows light microscope images of samples deposited under parameter sets #1 and #2. Set #1 achieved the highest density, while set #2 yielded the lowest density (see Table 3). For each set, the samples were prepared and analyzed both in longitudinal and in transverse cross-sections. The build direction is marked in Figure 8. It can be seen that set #2 resulted in high porous steel, namely elongated pores having average dimensions of 50 × 200 μm. These pores can be attributed to a lack-of-fusion due to insufficient energy input during the DED process. In this case, even unmelted powder particles in the pores could be noticed. In the case of set #1, some porosity can be observed. However, the size of pores is several microns. The origin of pores with such a small size could be residual porosity in the used gas-atomized powder [52]. It should be noted that these small pores were homogenously distributed in both cross-sectional orientations and are not expected to have a significant effect on the mechanical properties.

**Figure 9:**
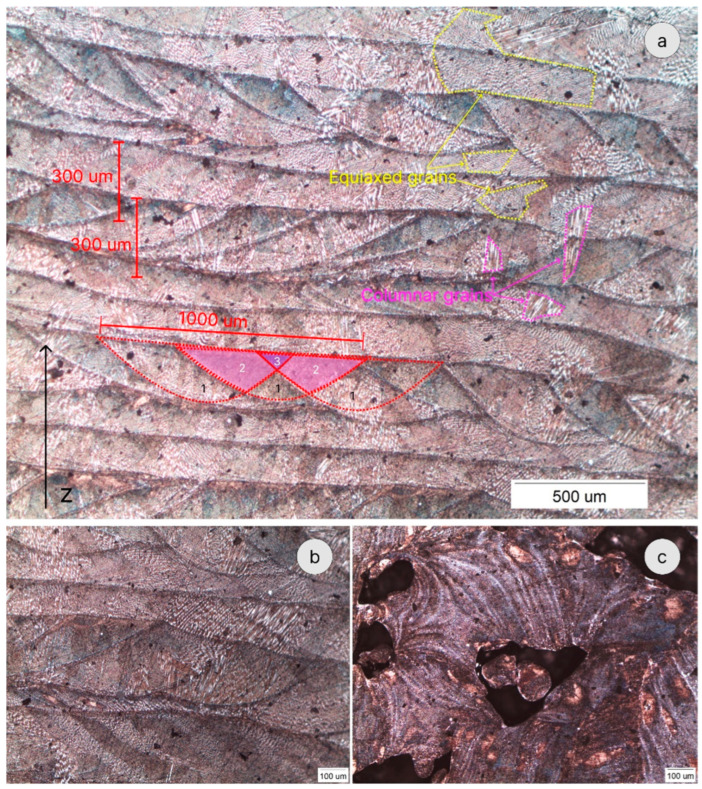
Light microscope images of chemically etched samples. (**a**,**b**) Various grain structures and melt pools overlapping in sample #1. (**c**) Typical porosity with unmelted powder particles in sample #2.

Figure 9 shows the typical microstructure of a DED’ed sample, showing the morphology of the melt pools and the layered structure. The grains exhibit the shape of the initial melt pool, and both equiaxed and columnar subgrains are present within the grains. This is in agreement with previously published data [12,21]. In Figure 9a, the numbers 1, 2, and 3 mark the melt pool zones that were affected (melted) once (1), 2 times (2), and three times (3), due to beads overlapping.

**Figure 10:**
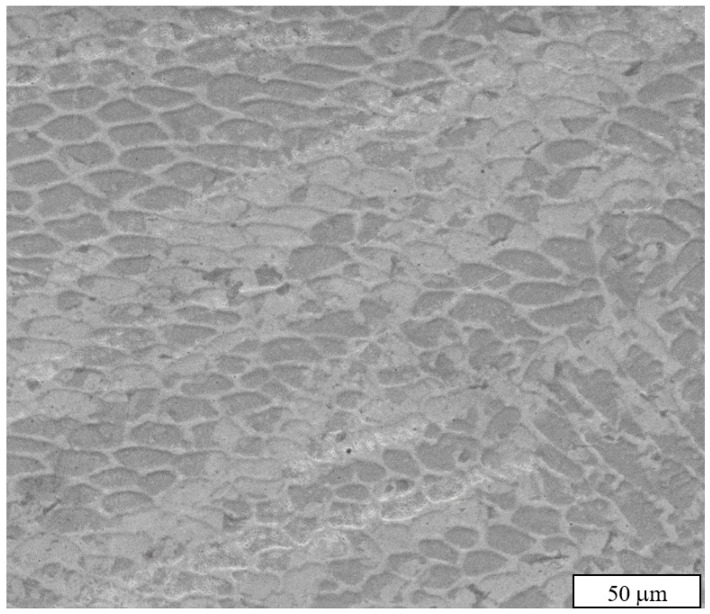
SEM image of cellular subgrain structure (~10 μm) of as-built sample #5.

**Figure 11:**
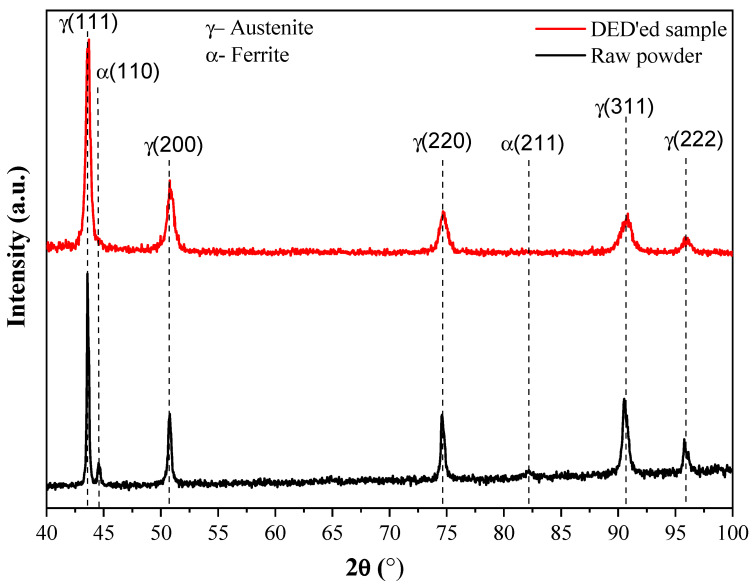
XRD patterns from a used powder feedstock and a DED’ed bulk sample.

Figure 10 shows that a fine cellular subgrain structure is present in nearly all builds, most notably in set #1 which had a GED value of 1.71 J/mm^3^. Attaining a fine-grain structure, as defined by the ASTM standard, appears to require rapid solidification rates and careful energy management [20]. This refined cellular subgrain structure exhibits consistent hardness, as experimentally determined (208–235 VHN), within the optimized process parameter combinations #1 and #3. Although it is conceivable that further optimization to enhance hardness could be explored, such investigation is beyond the scope of the present study. XRD patterns (Figure 11) show that although the used powder contains some α-Fe (ferrite), the DED’ed steel contains only γ-Fe (austenite). This can be explained by the high temperatures and high cooling rates in the DED process.

## 4. Conclusions

This study revealed the effect of the main process parameters (laser power, LSS, and PMFR) on the single-layer height, density, and microhardness of 316L SS samples additively manufactured by LENS^®^ DED. The response surface methodology (RSM) and central composite design (CCD) were employed to achieve this goal. Polynomial empirical prediction models correlating the applied processing parameters and the studied responses were developed. In DED, the layer thickness parameter in the STL file is actually the printing head movement along the *z*-axis. This is the reason why experimental single-layer trials are needed for proper calibration of the machine according to the specific material and part geometry.

1. This study found that a process parameter set with an energy input of 1.71 × 106 [J/((kg·s)·mm^3^)] resulted in fully dense steel with increased microhardness.
2. The microstructural analysis correlated with the prediction model’s density and microhardness outputs. The samples manufactured with optimal parameters (set #1) showed a low level of porosity.
3. Raising laser power and powder mass flow rate (PMFR) results in an increase in single-layer height, whereas an increase in laser scanning speed (LSS) under constant laser power and PMFR leads to a decrease in single-layer height.
4. PMFR exhibits the most substantial (negative) impact on the density response. This is anticipated given that an elevated PMFR value contributes to overgrowth, leading to the formation of pores and a lack of fusion.
5. For microhardness, an increase in PMFR results in microhardness reduction. Laser power demonstrates a comparatively lesser impact on microhardness, while an increase in LSS corresponds to an elevation in microhardness.

Additional analysis, including fatigue resistance and tensile testing, is planned for future research. Further research may include process parameter optimization of SS 316L parts according to geometry. This is motivated by the fact that thermal anisotropy is expected to lead to microstructural anisotropy in DED’ed parts.

In conclusion, this study successfully identified the optimal processing parameters and crucial dependencies for DED of 316L SS. Process parameter optimization and understanding the individual and complex synergistic effects of process parameters on the geometry, microstructure, and properties of the deposited material or part are essential for repeatable manufacturing of parts across a single or series of platforms over time or for minimizing defects such as porosity.

## Figures and Tables

**Table 2:** Chemical composition (wt.%) of the 316L powder according to supplier’s specification.

Fe	Cr	Ni	Mo	Mn	Si	P	C	S
Bal.	16.86	10.84	2.37	1.07	0.44	0.026	0.006	0.006

**Table 3:** CCD experimental matrix with the corresponding response values. *P*—laser power, *v*—LSS, *m*—PMFR, *h*—single-layer thickness, *ρ*—density, H—hardness. Samples #7 and #9 highlighted in grey are the two central points.

#	Pattern	Factors			Energy Density $10^{6}\left[J/\left(\frac{\mathrm{kg}}{s}{\mathrm{mm}}^{3}\right)\right]$	Responses		
		*P* [W]	*ν* [mm/min]	*m* [g/min]		*h* [mm]	*ρ* [g/cm^3^]	*H* [VHN]
1	0 0 −	475	400	10	1.71	1.53	7.92	232.3
2	− − +	250	200	30	0.6	4.19	7.32	163.3
3	+ + −	700	600	10	1.68	1.41	7.85	208.43
4	0 − 0	475	200	20	1.71	5.91	7.63	166.43
5	+ − −	700	200	10	5.04	6.27	7.87	177.1
6	− 0 0	250	400	20	0.45	1.95	7.67	189.17
7	0 0 0	475	400	20	0.86	5.02	7.57	177.27
8	+ 0 0	700	400	20	1.26	5.22	7.81	177.93
9	0 0 0	475	400	20	0.86	5.01	7.61	187.87
10	− − −	250	200	10	1.8	0.71	7.85	182.87
11	0 + 0	475	600	20	0.57	2.91	7.58	191.63
12	+ − +	700	200	30	1.68	6.90	7.86	171.43
13	− + +	250	600	30	0.2	2.58	7.62	161.12
14	+ + +	700	600	30	0.56	4.86	7.73	169.03
15	− + −	250	600	10	0.6	0.15	failed	failed
16	0 0 +	475	400	30	0.57	5.19	7.65	168

**Table 4:** DED processing parameters and their corresponding levels.

Factor	Units	Factor Level		
		−	0	+
Laser power (P)	W	250	475	700
LSS (v)	mm/min	200	400	600
PMFR (m)	g/min	10	20	30

**Table 5:** Summary of fit and lack-of-fit analyses.

Source	DF	Sum of Squares	Mean Square	*F*-Ratio
Single-layer height, R^2^ = 0.94, Adj-R^2^ = 0.85, RMSE = 0.7997 *				
Lack of Fit	4	0.02105004	0.005263	6.5781
Pure Error	1	0.00080000	0.000800	Prob > *F*
Total Error	5	0.02185004		0.2835
Density, *R*^2^ = 0.94, Adj-*R*^2^ = 0.83, RMSE = 0.0661 *				
Source	DF	Sum of Squares	Mean Square	*F*-ratio
Lack of Fit	4	0.02105004	0.005263	6.5781
Pure Error	1	0.00080000	0.000800	Prob > *F*
Total Error	5	0.02185004		0.2835
VHN, *R*^2^ = 0.89, Adj-*R*^2^ = 0.7, RMSE = 10.248 *				
Source	DF	Sum of Squares	Mean Square	F ratio
Lack of Fit	4	470.9853	117.746	2.1773
Pure Error	1	54.08000	54.080	Prob > *F*
Total Error	5	525.06535		0.4649

* RMSE—Root Mean Square error.

**Table 6:** The effect of different variables on the single-layer height response.

Term	Estimate	Std Error	*t*-Ratio		Prob > |t|
Laser Power (250,700)	1.508	0.25288	5.96	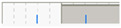	0.0010 *
PMFR (10,30)	1.365	0.25288	5.40	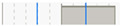	0.0017 *
LSS (200,600)	−1.207	0.25288	−4.77	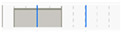	0.0031 *
Laser Power × LSS	−0.59125	0.282728	−2.09	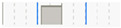	0.0815
PMFR × PMFR	−0.803103	0.492506	−1.63	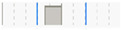	0.1541
Laser Power × Laser Power	−0.578103	0.492506	−1.17	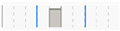	0.2850
Laser Power × PMFR	−0.22875	0.282728	−0.81	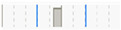	0.4494
LSS × PMFR	0.22125	0.282728	0.78	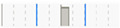	0.4636
LSS × LSS	0.2468966	0.492506	0.50	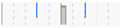	0.6340

* Denotes a statistically significant value.

**Table 7:** The effect of different variables on the density response.

Term	Estimate	Std Error	*t*-Raio		Prob > |t|
PMFR (10,30)	−0.143954	0.025513	−5.64	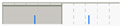	0.0024 *
Laser Power × PMFR	0.113692	0.029672	3.83	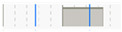	0.0122 *
Laser Power × LSS	−0.091192	0.029672	−3.07	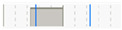	0.0277 *
PMFR × PMFR	0.1241139	0.04141	3.00	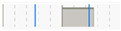	0.0302 *
Laser Power (250,700)	0.0530464	0.025513	2.08	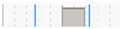	0.0922
Laser Power × Laser Power	0.0791139	0.04141	1.91	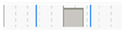	0.1143
LSS (200,600)	0.0379536	0.025513	1.49	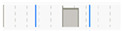	0.1970
LSS × LSS	−0.055886	0.04141	−1.35	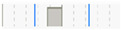	0.2350
LSS × PMFR	−0.011192	0.029672	−0.38	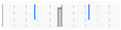	0.7215

* Denotes a statistically significant value.

**Table 8:** The effect of different variables on the microhardness response.

Term	Estimate	Std Error	*t*-Raio		Prob > |t|
PMFR (10,30)	−21.33406	3.954894	−5.39	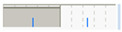	0.0030 *
LSS (200,600)	11.462059	3.954894	2.90	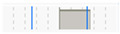	0.0339 *
LSS × PMFR	−12.32007	4.59975	−2.68	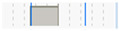	0.0439 *
PMFR × PMFR	12.326582	6.419319	1.92	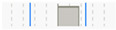	0.1129
Laser Power × PMFR	7.3625738	4.59975	1.60	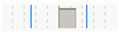	0.1704
LSS × LSS	−8.793418	6.419319	−1.37	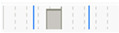	0.2291
Laser Power (250,700)	−3.808059	3.954894	−0.96	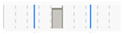	0.3798
Laser Power × LSS	−3.945074	4.59975	−0.86	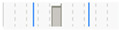	0.4303
Laser Power × Laser Power	−4.273418	6.419319	−0.67	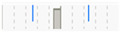	0.5351

* Denotes a statistically significant value.

## Data Availability

Data are contained within the article.
